# Structural insight into a CE15 esterase from the marine bacterial metagenome

**DOI:** 10.1038/s41598-017-17677-4

**Published:** 2017-12-08

**Authors:** Concetta De Santi, Osman ABSM Gani, Ronny Helland, Adele Williamson

**Affiliations:** 1 Department of Chemistry, UiT The Arctic University of Norway, Tromsø, N-9037 Norway; 2 NorStruct, Department of Chemistry, UiT The Arctic University of Norway, Tromsø, N-9037 Norway

## Abstract

The family 15 carbohydrate esterase (CE15) MZ0003, which derives from a marine Arctic metagenome, has a broader substrate scope than other members of this family. Here we report the crystal structure of MZ0003, which reveals that residues comprising the catalytic triad differ from previously-characterized fungal homologs, and resolves three large loop regions that are unique to this bacterial sub-clade. The catalytic triad of the bacterial CE15, which includes Asp 332 as its third member, closely resembles that of family 1 carbohydrate esterases (CE1), despite the overall lower structural similarity with members of this family. Two of the three loop regions form a subdomain that deepens the active site pocket and includes several basic residues that contribute to the high positive charge surrounding the active site. Docking simulations predict specific interactions with the sugar moiety of glucuronic-acid substrates, and with aromatically-substituted derivatives that serve as model compounds for the lignin-carbohydrate complex of plant cell walls. Molecular dynamics simulations indicate considerable flexibility of the sub-domain in the substrate-bound form, suggesting plasticity to accommodate different substrates is possible. The findings from this first reported structure of a bacterial member of the CE15 family provide insight into the basis of its broader substrate specificity.

## Introduction

The CAZymes family 15 carbohydrate esterases (CE15s) include members with glucuronoyl esterase activity that are predicted to act on the ester linkages between the 4*-O-*methyl-glucuronoyl substitutions on xylan of hemicellulose and the aromatic alcohol of lignin as their natural substrate; the so-called lignin-carbohydrate-complex (LCC). The majority of characterized CE15s are specific for esters of 4*-O-*methyl-D-glucuronic acid (MeGlcA) and derive from fungi^1–6^. However, two bacterial versions display broader substrate scopes: a dual-specificity enzyme from *Ruminococcus flavefaciens* which has acetyl xylan activity, likely imparted by an appending domain^7,8^, and the single-domain CE15 ‘MZ0003′ which was cloned from a marine metagenomic library and has activity against a broad range of acetyl esters^9^. Recent analysis of CE15 enzymes using peptide pattern recognition (PPR), a non-alignment-based method, supports separation of MZ003 into a separate clade to the fungal CE15s, and has identified additional, more distantly-related, CE15 members^10^. This expanded family clusters into 24 groups, three of which are predominantly fungal, one contains higher plant sequences and the remainder are bacterial.

Two crystal structures of CE15s, both from fungi, are available to date: the catalytic domain of the Cip2 enzyme from *Hypocrea jecorina* (synonym *Trichoderma reesei*, PDB 3pic)^11^, and the glucuronoyl esterase StGE2 of the thermophilic *Myceliophthora thermophila* (synonym *Sporotrichum thermophile*, PDB 4g4g, PDB 4g4j)^12^. Both have the three-layer αβα-sandwich typical of serine-type hydrolases, and include the catalytic serine in a novel consensus G-C-S-R-X-G, confirmed by mutagenesis as well as structural studies^4^. The catalytic triad which comprises Ser, His and Glu is relatively surface-exposed which is consistent with the proposed role of the enzyme in cleaving bonds between the bulky hemicellulose and lignin moieties. Rigidity of the catalytic site is maintained by a disulfide bond at the entrance of the active site. Co-crystallization of an inactive StGE2 mutant with methyl 4-*O*-methyl-D -glucopyranuronate revealed the active-site cavity residues responsible for interaction with the uronic acid part of the substrate including Lys, Gln and Trp^12^.

Consistent with the bacterial MZ0003 having a different substrate specificity to fungal CE15s, significant structural differences were predicted based on sequence alignments^9^. This includes the lack of Glu in the conserved position of the catalytic triad, and the presence of three regions that are not found in the fungal enzymes, and were modelled as loops. To discover the real conformation of these non-homologous regions, as well as determine the catalytic site residues, we have solved the crystal structure of MZ0003 to 1.9 Å. This represents the first structure of a bacterial CE15 enzyme, and is the third and most divergent CE15 structure reported. In an attempt to understand the substrate specificity of MZ0003, and obtain clues to its native substrate, we docked several model substrates that are known to be hydrolysed by *in vivo* ^9^ and several substrate mimics of LCCs which are hydrolysed by other CE15 enzymes^5^ into this structure. We predicted Asp 332 as the catalytic residue which was further confirmed by site directed mutagenesis studies. We also used molecular dynamics simulations to study the overall stability of the docked substrate into MZ0003.

## Results

### Overall structure of MZ0003

The asymmetric unit of the MZ0003 crystal structure contains only one molecule, however a dimer can be generated based on crystallographic symmetry, yielding a quaternary structure resembling that observed in the homolog 3pic (Supplementary Figure 1). The dimer interface consists of about 20 hydrogen bonds, two salt bridges (Glu 61 – Lys 199), hydrophobic interactions and water-mediated interactions. The loop/helix region consisting of residues 187–206 extends from one monomer forming a “hook” which is embedded in the second molecule of the MZ0003 dimer. This is in contrast to the helix regions in 4g4j and 3pic, which are packed closer to the molecule surface. The electron density is well defined in this region, demonstrating that this is not an artifact of poorly defined density due to flexibility. The possibility that this quaternary structure is induced under crystallization can, however, not be ruled out, as MZ0003 was previously indicated by both gel filtration and native PAGE to be a monomer with extended conformation in solution^9^.

None of the six cysteine residues of MZ0003, which are all found in the conserved core regions of the protein, are involved in disulphide bonds in the crystal structure. Denaturing electrophoresis also indicates that disulphide bonds are not present in the solution structure of the protein as no difference in molecular weight was detected in the presence or absence of reducing agent (data not shown).

The structure coordinates of MZ0003 have been submitted to the protein data bank with the PDB identifier 6ehn. Residue numbering in both the PDB file and this publication omits the predicted 25 residue N-terminal signal sequence, as the mature form of MZ0003 was used for both crystallization and biochemical assays, with the first residue of the mature protein being designated as number 1.

### A novel sub-domain forms a deep catalytic pocket in MZ0003

**Figure 1:**
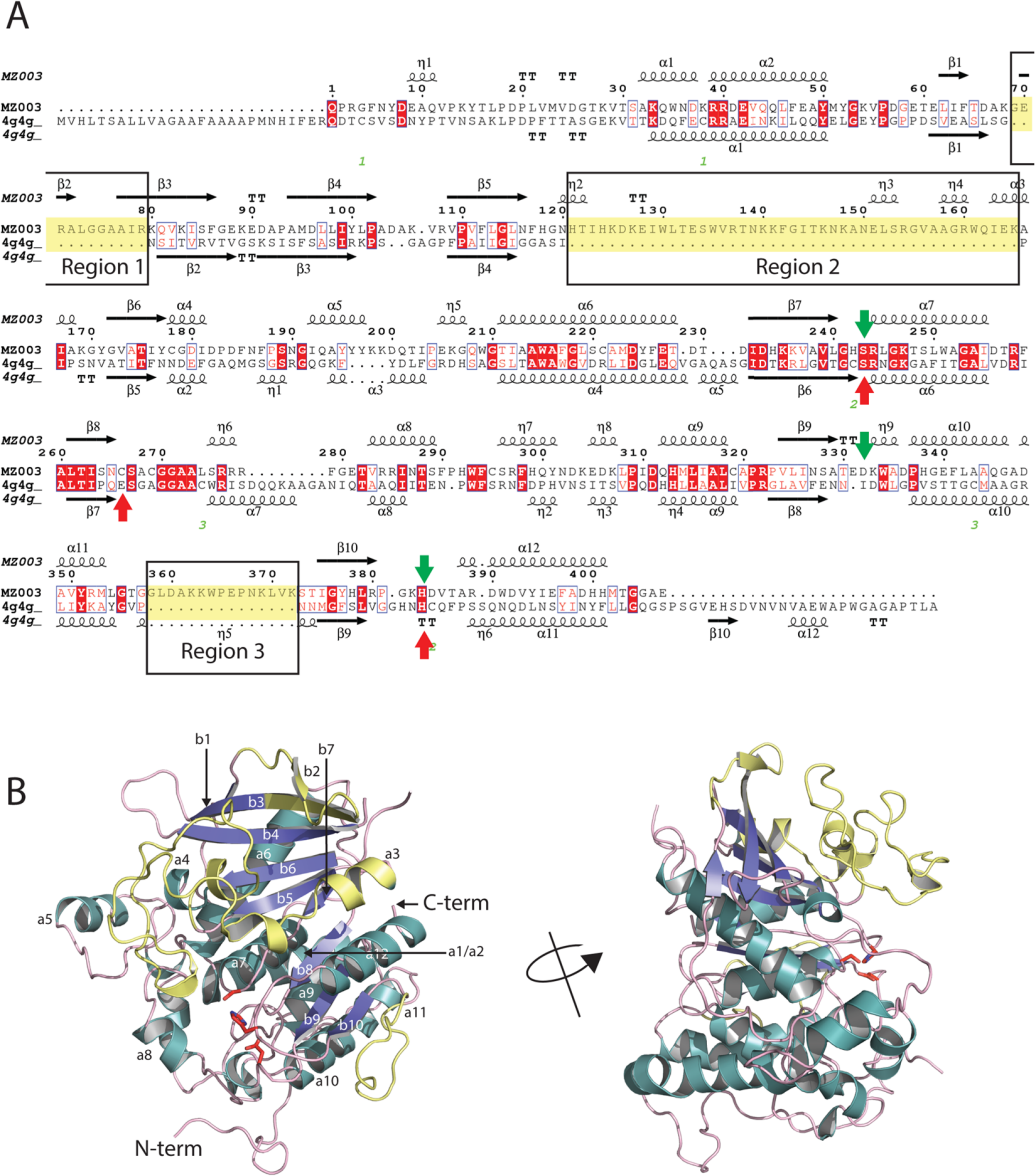
Structure-based sequence alignment (**A**) of MZ0003 with 4g4g, a CE15 from *M*. *thermophilia*. Fully conserved residues are highlighted in red; residues with conserved properties are indicated in red text. Residues of the catalytic triad are indicated with green and red arrows for MZ0003 and 4g4g respectively. Loop regions identified in MZ0003 which are not conserved in fungal CE15s are boxed and indicated in yellow. (**B**) Overall fold of the MZ0003 monomer. Novel loop regions are colored yellow, the side chains of the catalytic residues are shown in red.

MZ0003 displays the greatest structural similarity to the fungal CE15 glucuronyl esterases from *H*. *jecorina* (PDB 3pic) and *M*. *thermophilia* (PDB 4g4g), and has detectable homology with various other bacterial esterases (Table 1). MZ0003 differs from the canonical α/β hydrolase fold in essentially the same ways as the fungal CE15s 4g4g and 3pic with additional β-strands extending the central β-sheet at the N-terminus, and a larger number of α- and 3_10_ helices sandwiching it on both sides. The structure-based sequence alignment MZ0003 and PDB 4g4g are shown in Fig. 1(A).

**Table 1:** Structural homologs of MZ0003 aligned using PDBefold. The Z-score is the significance of the alignment based on Gaussian statistics; Root-mean-square deviation (RMSD) is calculated between Cα atoms; % sequence identity (seq.i.d.) is the percentage of structurally aligned positions with identical residues.

Protein	Species	PDB	Z score	RMSD	seq. i.d. (%)
Glucuronoyl esterase	*Hypocrea jecorina*	3pic	10.6	1.91	29
Glucuronoyl esterase	*Myceliophtora thermophilia*	4g4g	10.8	1.77	26
Dienelactone hydrolase	*Anabaena variabilis*	2o2g	9.3	2.21	17
C-terminal esterase domain of lc-est1	*Metagenome*	3wyd	8.7	2.31	20
tt1662	*Thermus thermophilus*	1ufo	8.7	2.46	11
Cinnamoyl esterase lj0536	*Lactobacillus johnsonii*	3pf8	8.6	2.38	11
est1e	*Butyrivibrio proteoclasticus*	2wtm	9.3	2.30	14
Putative dienelactone hydrolase	*Klebsiella pneumoniae*	3f67	9.3	2.54	13
Alkaline esterase	Marine sediment metagenome	4rgy	7.2	2.54	16

**Figure 2:**
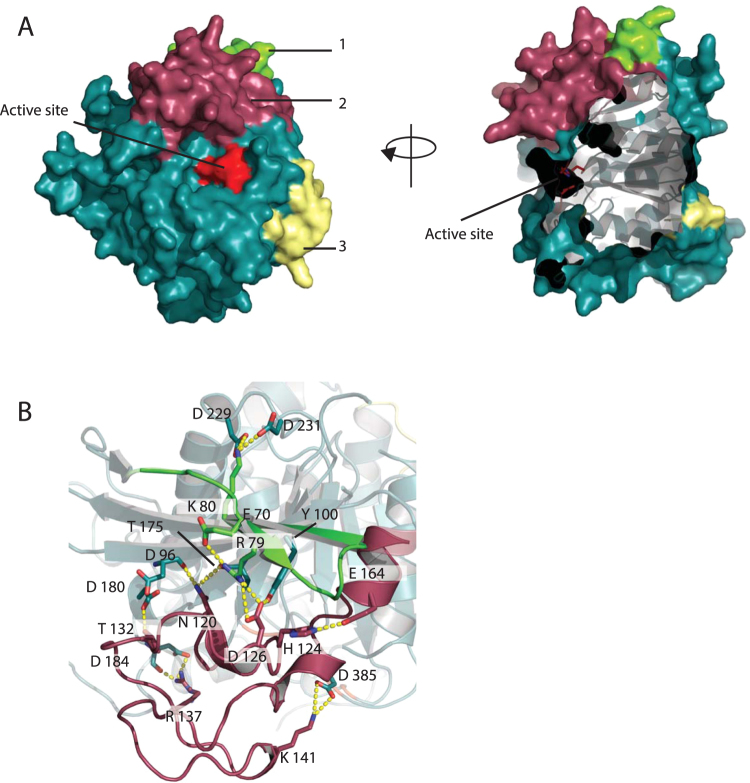
Surface view (**A**) of MZ0003 with the loop regions 1-3 coloured green, dark pink and yellow. The protein core is coloured teal, and the catalytic triad is bright red. (**B**) Salt bridge and hydrogen bond network between regions 1 and 2, contributing to the novel subdomain.

The overall topology of MZ0003 reveals the presence of three large inserts, referred to as regions 1, 2 and 3, which do not have counterparts in either of the fungal homologs (Fig. 2A, Supplementary Figure 2). Region 1, located between β1 and β3 of the central sheet is 11 residues in length. The first 8 residues comprise an unstructured loop and a small (three residue) β strand (β2), while the last three residues extend the N-terminal end of β3. Region 2 is a 45 residue loop between β5 and β6 which includes three 3_10_ helices and a short (6 residue) α-helix (α6). Together, regions 1 and 2 form a subdomain at the N-terminal end of the central β-sheet, with the helices of region 2 on the same side as the catalytic residues and the loops of region 2 protruding over the active site and partially occluding it (Fig. 2A). Interactions between region 1 and the conserved core include the β-sheet interactions between the N-terminus of β3 and the C-terminus of β4, as well as a salt bridge between the side chain of Lys 80 in the middle of β3 and a pair of Asp (Asp 229 and 231) in the loop between α6 and β7 (Fig. 2B). Contacts between region 2 and the protein core include two hydrogen bonds to β4 (side chain N of Asn 120 to the main chain carbonyl oxygen of Asp 96, and side chain of Asp 126 to side chain of Tyr 100), a hydrogen bond between the side chain of Thr 132 and Asp 180 of α4, a hydrogen bond between the side chain of Arg 137 and the carbonyl oxygen of Asp 186. There is also a salt bridge between Lys 141 and Asp 385. Asp 385 is adjacent to the catalytic His 384, providing a link between this lid domain and the catalytic residues. It is interesting to note that in fungal homologs Cys 346 occupies this position adjacent to the catalytic His, and is involved in a disulfide bond with Cys 211 which is directly adjacent to the catalytic serine, and enhances rigidity in the *M*. *thermophila* enzyme catalytic site. It is likewise possible that the salt bridge in MZ0003 between the lid and the active site loop couples substrate binding to activity. The N- and C-terminal ends of the region 2 insert are connected by a salt bridge between His 124 and Glu 164. Regions 1 and 2 are connected by a salt bridge between Asp 126 of region 2 and the NH2 of Arg 79 of β3 in region 1. The residues involved in the salt bridges are partially conserved in bacterial homologs. The third insertion is a 17 residue loop which is essentially untethered to the protein core.

**Figure 3:**
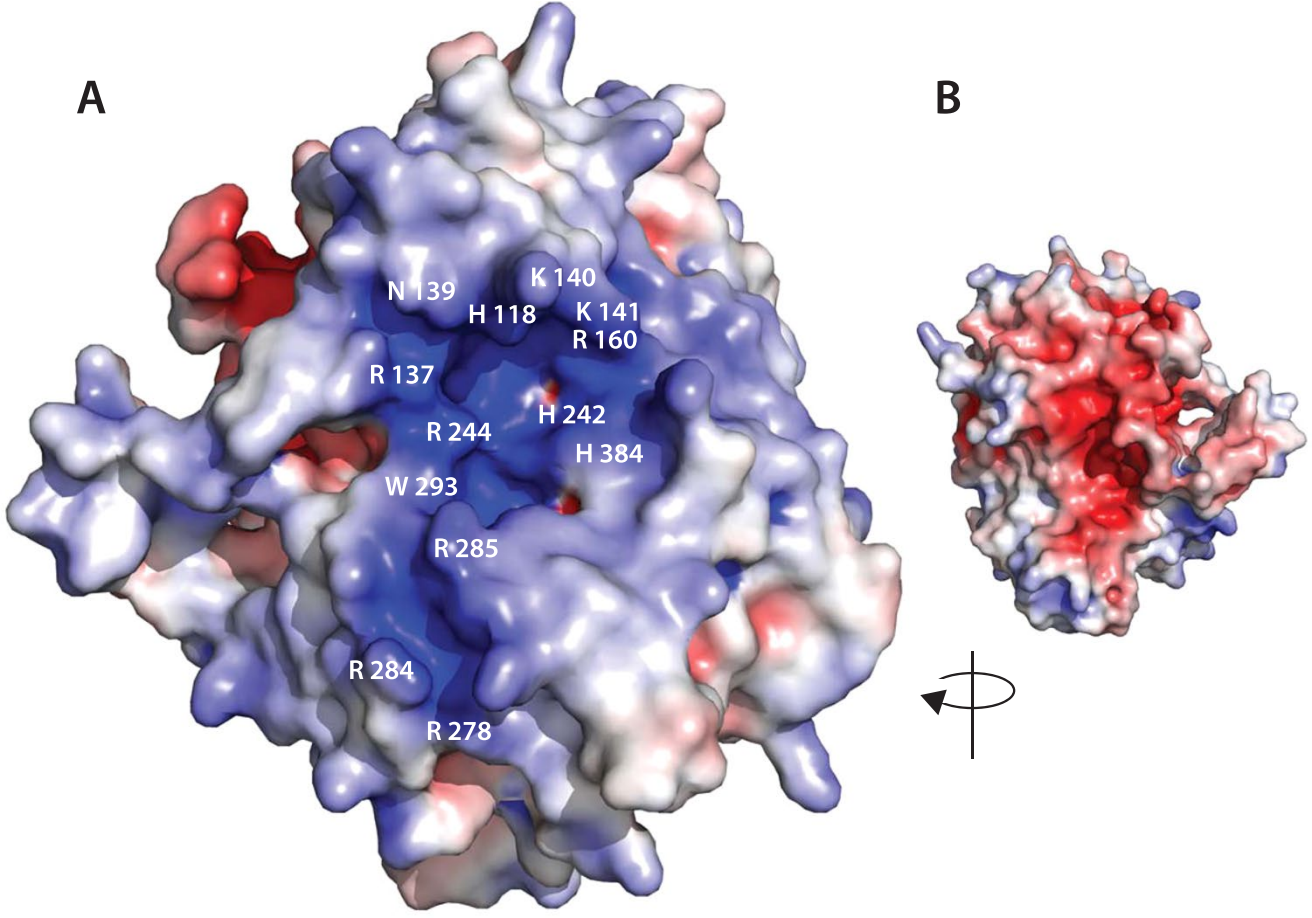
MZ0003 colored by electrostatic surface potential. The surface potential was generated using APBS^35^, with positively charged areas shown in blue and negatively charged areas in red. (**A**) Substrate-binding face of MZ0003. Residues contributing to the positive charge of the catalytic cleft, or predicted to interact with methyl 4*-O-*methyl-D-glucopyranuronate by docking simulation (below) are labeled (**B**) Opposite face of MZ0003.

In addition to narrowing the access to the active site, region 2 includes several basic residues, namely Arg 137, Asn 139, Lys 140 and 141, Arg 160, which contribute to the positively charged surface potential surrounding the catalytic pocket (Fig. 3A). This charge distribution is markedly asymmetrical, with the opposite face of the enzyme having a calculated overall negative charge (Fig. 3B).

## Catalytic Site

**Figure 4:**
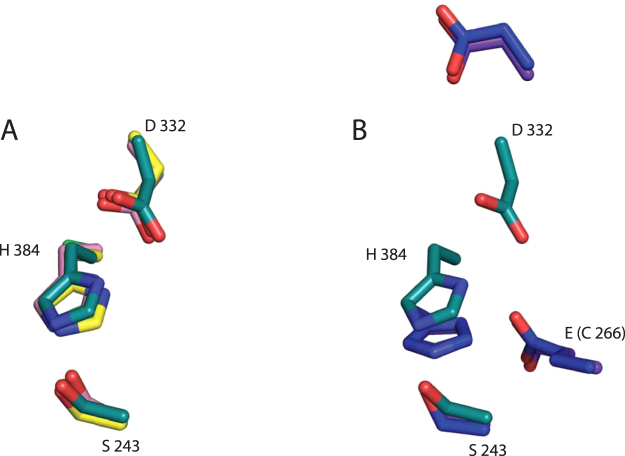
(**A**) Active site residues of MZ0003 (teal) superimposed onto the catalytic triads of the feruloyl esterase *of B*. *proteoclasticus* (pink) and the TT1662 esterase from *T*. *thermophilus* HB8 (yellow). (**B**) MZ0003 (teal) superimposed onto the catalytic triads of *M*. *thermophilia* StGE2 (blue) and *H*. *jecorina* Cip2 (purple- overlaped by StGE2).

The catalytic serine and histidine residues (Ser 243, Ser 278, Ser 213 and His 384, His 411, His 346 in MZ0003, 3pic and 4g4g, respectively) are well conserved in the three structures and were previously confirmed by mutagenesis in MZ0003^9^. However, the position of the third catalytic residue, Glu 301 in 3pic and Glu 236 in 4g4g is occupied by Cys 266 in MZ0003. The geometric arrangement in the active site indicates that Asp 332 is the third residue of the triad in MZ0003, functioning to adjust the basic character of His 343. This was confirmed as the Asp332Ala mutant of MZ0003 is inactive in assays with both *p-*nitrophenyl acetate (*p-*NP acetate) and methyl 4-*O*-methyl-D-glucopyranuronate (data not shown). Interestingly, the catalytic residues of MZ0003 map almost perfectly onto the triads of more distantly related homologs TT1662 from *Thermus thermophilus HB8* ^13^, cinnamoyl esterase of *Lactobacillus johnsonii* ^14^ and the feruloyl esterase (Est1E) from the rumen bacterium *Butyrivibrio proteoclasticus* ^15^ (Fig. 4). The position equivalent to MZ0003 Asp 332 is occupied by isoleucine in both 3pic and 4g4g.

Electron density found in the active site, is consistent with a molecule of glycerol and interacts with the catalytic Ser 243 OD (2.78 Å), the side chain of Glu 281 (bidentate interactions O2 – OE1 of 2.42 Å and O3 – OE2 of 2.56 Å) and Lys 247 (2.70 Å). The glycerol occupies the same conformational space as the methyl 4*-O-*methyl-D-glucopyranuronate molecule in the catalytically inactive mutant of *M*. *thermophilia* StGE2 (PDB 4g4j), and may explain why attempts to soak substrate into the active sites of both wild-type and inactive mutants of MZ0003 were unsuccessful, and is consistent with our observation that addition of 50% glycerol during protein storage significantly decreases activity.

Five Zn ions were identified in the electron density maps of the heavy atom structure of MZ0003. One was bound tightly to the catalytic residues His 384 (2.01 Å) and Ser 243 (2.11 Å), and a second Zn was bound to His 118 (2.18 Å) close to the catalytic residues, and these probably explain the inhibitory effect of Zn observed in previous catalytic assays^9^.

## Docking and Dynamics

**Figure 5:**
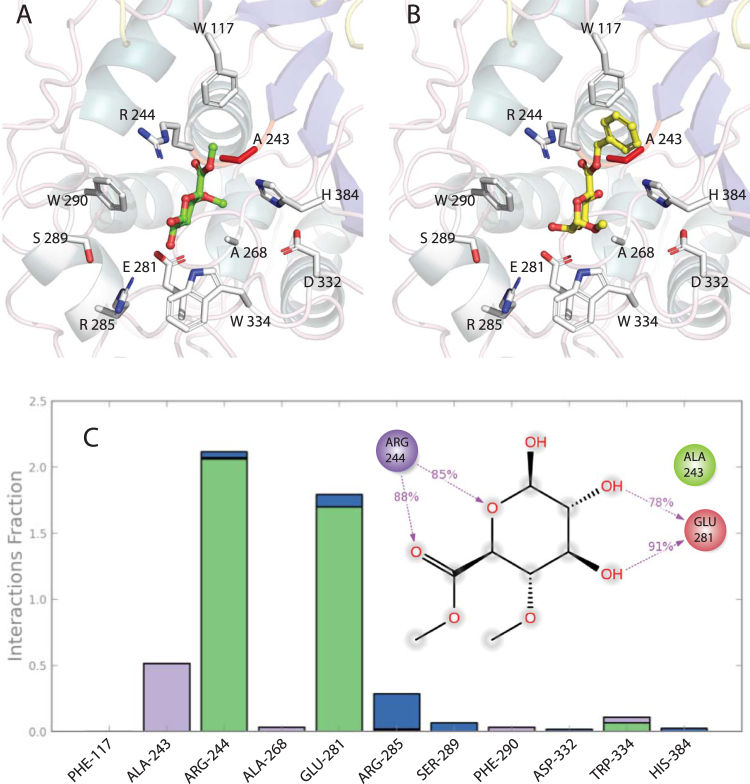
Structure of MZ0003 with (**A**) Methyl 4*-O-*methyl-D-glucopyranuronate and (**B**) benzyl (methyl a-D-glucopyranoside) uronate docked into the catalytic site. (**C**) ligplot diagram and plot showing predicted fraction of interactions between methyl 4*-O-*methyl-D-glucopyranuronate and MZ0003 during a 10 ns MD simulation in 0.5 M NaCl. Green indicates hydrogen bonds, violet hydrophobic interactions, and blue ionic bonds.

Structure coordinates of MZ0003 where the catalytic Ser 243 was changed to Ala were used for docking simulations with ester substrates (Table 2), some of which have been tested *in vivo* with MZ0003 previously^9^. The best docking score of −7.125 K cal mol^−1^ was obtained for the model substrate methyl 4*-O-*methyl-D-glucopyranuronate with key interactions involving MZ0003 side chains of Arg 244, Glu 281 and Arg 285 (Fig. 5A). A series of model compounds previously synthesized by d’ Errico and coworkers to mimic the LCC^5^ also gave significant scores for predicted binding to MZ0003 with similar interactions as methyl 4*-O-*methyl-D-glucopyranuronate, and additional pi-pi stacking between the Phe 117 side chain the ligand phenyl ring (Fig. 5B, and Supplementary Figure 3A–C). Further simulations of a Benzyl (methyl 4*-O-*methyl-a-D-glucopyranoside) urinate derivative structure with methoxy and hydroxyl groups on positions 4 and 5 of the aromatic ring predicts a further electrostatic interaction between the ether oxygen and the side chain of Arg 160 (Table 2, Supplementary Figure 3D). Binding energies are very similar for substrates that include a 4*-O-*methyl substituent on the sugar moiety (i.e. methyl 4*-O-*methyl-D-glucopyranuronate and benzyl (methyl 4*-O-*methyl-α-D-glucopyranoside) urinate) and substrates that lack this modification (i.e. methyl-D-glucopyranuronate, benzyl (methyl α -D-glucopyranoside) uronate). Significantly lower binding energies are predicted for *p-*nitrophenyl ester compounds than for uronic-acid esters, especially in the case of the larger *p-*nitrophenyl octanate molecule.

**Table 2:** Binding energies (ΔG_bind_ **)** for substrates docked onto MZ0003 S243A.

Substrate	ΔG_bind_ (K cal mol^−1^)	Hydrolysed by MZ0003
Methyl 4*-O-*methyl-D-glucopyranuronate	−7.125	+^a^
Methyl-D-glucopyranuronate	−7.046	+^a^
Benzyl-D-glucopyranuronate	−6.750	+^a^
Benzyl (methyl 4*-O-*methyl-a-D-glucopyranoside) urinate	−6.623	nd^b^
Benzyl (methyl α-D-glucopyranoside) uronate	−6.438	nd^b^
Phenyl (methyl α-D-glucopyranoside) uronate	−6.259	nd^b^
Phenylpropyl (methyl a-D-glucopyranoside) uronate	−5.050	nd^b^
Allyl-D-glucopyranuronate	−4.931	+^a^
*p-*nitrophenyl acetate	−4.165	+^a^
*p-*nitrophenyl octanate	−1.640	−^a^

^a^Reference for activity assays^9^.

^b^Compound described in^5^.

In order to confirm the binding mode and stability of the substrate in the enzyme binding pocket, 10 ns molecular dynamics simulations were carried out on the docked complex between MZ0003 and methyl 4*-O-*methyl-D-glucopyranuronate. This was done at both 0.5 M NaCl, the natural salinity of an extracellular marine enzyme, and 1.0 M NaCl, the NaCl concentration where the best activity was previously measured^9^. The simulation had converged by 5 ns with both the ligand and the protein remaining stable, and a protein root mean squared deviation (RMSD) less than 2.0 Å throughout the time course. The profiles were similar with both 0.5 M and 1 M NaCl, although the RMSD for both the protein and ligand were lower with at the higher salt concentration (data not shown). The plot of RMSD along the protein chain indicated that the N-terminal half was much more flexible, with the greatest fluctuations between positions 120–150, and 170–200, which correspond to the N-terminal half of the lid region 2, and β6 – α5 of the protein core (Supplementary Figure 4). The fraction of interactions between specific protein side chains and substrate chemical moieties highlights the importance of Arg 244 which is in contact with both the hemiacetyl oxygen of the glucopyranuronate ring and the carbonyl oxygen of the ester group, and Glu 281 which contacts the peripheral hydroxyl groups of the sugar.

## Discussion

Analysis of the crystal structure of the bacterial CE15 MZ0003 reveals significant differences in the active-site region compared to fungal CE15 enzymes. This is in spite of MZ0003 sharing the highest overall structural similarity with fungal CE15s, and possessing the G-C-S-R-X-G motif characteristic of the CE15 class, rather than the classical G-x-S-x-G consensus sequence of other carboxy esterases^16^. The present work, coupled with our earlier mutagenesis studies^9^ show that the catalytic triad residues of MZ0003 are almost identical in both the nature of the amino acid, and their position in the three dimensional protein structure to carboxyesterases with which MZ0003 shares lower overall similarity, including dienelactone, cinnamoyl and feruloyl hydrolases^14,15^.

Although the biological substrate of MZ0003 remains unknown, some insight can be gained from analysis of the substrate binding pocket. Compared to the surface-exposed active sites of fungal CE15s the substrate pocket of MZ0003 is deepened by a 58 amino acid subdomain that also contains several basic amino acids contributing to a positively charged binding cleft. The relatively occluded access to the catalytic residues, suggests that MZ0003 may act on smaller or fragmented substrates rather than the intact LCC polymers that are the proposed target of the fungal enzymes. Molecular dynamics simulations with the small LCC model compound methyl 4*-O-*methyl-D-glucopyranuronate bound predict significant flexibility in the insert region comprising the sub-domain, suggesting plasticity in this region which could accommodate larger molecules. This plasticity may also explain the previously-determined broad substrate range of MZ0003, which besides glucoronate derivatives, includes acetyl esters of a variety of aromatic compounds and sugars^9^.

In addition to predicting binding of MZ0003 to methyl 4*-O-*methyl-D-glucopyranuronate, which it was previously shown to hydrolyze^9^, docking studies indicate that specific interactions may be formed with larger aromatic substrates, including those bearing hydroxy and methoxy functionalities. A particularly interesting interaction is the predicted pi-pi stacking between the Phe 117 side chain of MZ0003 and the phenyl ring of LCC model compounds that are known to be hydrolyzed by fungal CE15s^5^. In the natural LCC, these aromatic rings of the lignin alcohol bear methoxy and hydroxyl functional groups^17^, which would provide further opportunities for interaction with the basic side chains lining the catalytic pocket of MZ0003. Indeed, our docking study with methoxy and hydroxylated derivatives of these compounds predicts specific interactions with side chains of the sub-domain region. Interestingly, docking scores were similar between methyl 4*-O-*methyl-D-glucopyranuronate and methyl-D-glucopyranuronate, which lacks the 4*-O-*methyl substituent suggesting that, consistent with previous experimental findings, this position is not critical for substrate binding by MZ0003^9^. The significantly lower binding energy of *p-*NP acetate in comparison to these more complex substrates suggests that the hydrolysis previously demonstrated by MZ0003^9^ is a consequence of promiscuity by the enzyme rather than specific active-site interactions. However, the occluded active site of MZ0003 may favour hydrolysis of *p-*NP acetate once it has diffused in, relative to the surface-exposed catalytic triads of fungal CE15s. The extremely poor docking score of *p-*nitrophenyl octanate is consistent with the complete lack of MZ0003 activity with this substrate^9^, and validates this *in silico* approach as an accurate way to predict potential substrate specificities for MZ0003.

In our initial characterization of the biochemical properties of MZ0003, we postulated that an algal polymer could serve as a substrate for this enzyme due to the scarcity of LCC material in ocean sediments where MZ0003 derives from^9^. Other plausible substrates are humic or fulvic acid substances which are discharged into the oceans from freshwater streams and are degraded by microbes in the esturine and marine environment^18,19^. Like lignin, these acids bear aromatic substituents that are predicted to form favourable stacking interactions with the MZ0003 active site.

In summary, the deeper, positively charged substrate binding pocket which is formed by a novel sub-domain in MZ0003 is likely responsible for its broader substrate specificity relative to fungal homologs, and indicates diversity of activity may exist among unexplored clades of CE15 enzymes.

## Methods

### Crystallization

An in-house screen of 96 conditions were tested to determine initial crystallization conditions by sitting-drop vapor-diffusion method using a Phoenix crystallization robot (Art Robbins Instruments) and MRC plates with a reservoir volume of 60 µl per well. The predicted mature form of MZ0003 without the 25 amino acid signal peptide was prepared as described previously^9^, concentrated up to 12 mg/ml in 50 mM Tris HCl pH 8.0; 5% glycerol; 750 mM NaCl, and drop solutions were prepared by mixing 0.5 µl well solution and 0.5 µl protein solution. The plate was stored at 4 °C and crystals appeared after 1 month. The largest crystals for X-ray diffraction were grown at 4 °C to a size of approximately 0.2 × 0.2 × 0.1 mm^3^ using the hanging drop method with a 200 µl reservoir solution containing 0.1 M Na-citrate pH 6.0 and PEG3350 24%. Heavy atom derivatives were obtained by soaking native crystals in a solution of 20 mM ZnCl_2_ with 24% PEG 3350 and 0.1 M succinate pH 5.6, for 2 hours or overnight. Crystals for data collection were cryo protected using the heavy atom solution containing 10% glycerol.

### Data collection and structure determination

Native data was collected at Bessy BL 14.1 to 1.9 Å. Heavy atom derivative data to 2.1 Å was collected at the European Synchrotron Radiation Facility (ESRF) ID23-1 just above the Zn edge at 1.28202 Å. Data collection statistics is listed in Table 3. All data was integrated and scaled using XDS^20^ and AIMLESS^21^. The structure was solved by SAD using the AutoSol option in PHENIX^22–28^. 289 of 408 residues were automatically built in electron density. Manual inspection of the electron density maps allowed fitting of additional 85 residues and five Zn ions into electron density. The SAD structure was subsequently used as search model in molecular replacement^29^ using the high-resolution data. Manual inspection of electron density maps and model building using Coot^30^, followed by positional refinement in REFMAC^31^, allowed building of a final model consisting of continuous electron density from residue 5 to 403 with the exception of residues 105-106. Data collection and refinement statistics are listed in Table 3. The final model was deposited in the Protein Data Bank as entry 6ehn

**Table 3:** X-ray crystallographic data-collection and refinement statistics for MZ0003.

	Heavy atom derivative	Native
**PDB code** 6ehn		
**Data collection**		
Beam line	ESRF, ID 23-1	Bessy, BM 14.1
Data collection wavelength (Å)	1.28202	0.91840
Diffraction limit (Å)	2.1	1.90
Space group	*P*3_1_21	*P*3_2_21
Outer shell values (Å)	2.16–2.1	1.94–1.9
Unit cell parameters		
a-axis (Å)	107.71	110.21
b-axis (Å)	107.71	110.21
c-axis (Å)	76.76	78.67
Total no. of reflections	562565 (28153)	243936 (15636)
No. of unique reflections	30304 (2407)	43689 (2774)
Completeness (%)	99.9 (98.5)	99.9 (100.0)
I/σ(I)	6.8 (0.8)	10.1 (1.3)
Mean I/σ(I)	26.8 (2.6)	16.4 (3.0)
R_merge_ (%)	6.6 (93.9)	6.2 (54.2)
R_pim_ (%)	2.2 (40.9)	4.2 (36.6)
Multiplicity	18.6 (11.7)	5.6 (5.6)
Wilson B (Å^2^)	39.6	25.48
**Refinement**		
R_work_ (%)	20.36	18.32
R_free_ (%)	25.68	22.29
Average *B* factors (Å^2^)	49.17	33.04
No. protein atoms	2983	3133
No. other atoms		
Solvent	120	315
Glycerol	0	1
Zn	5	0
R.m.s. deviations		
Bond lengths (Å)	0.019	0.019
Bond angles (°)	2.056	1.934
% residues in regions of the Ramachandran plot		
Most favoured	92.8	94.4
Additionally allowed	5.3	4.1
Outlier	1.9	1.5
DPI (based in Rfree)	0.2389 (0.2080)	0.1247 (0.1240)

### Mutagenesis and assay of the D332 mutant

Site-direct mutagenesis method was used to generate an MZ0003 mutant by using the QuickChange Site-Directed Mutagenesis kit (Agilent Technologies) according to the manufacturers’ instructions. Mutagenic primers with a single amino acid substitutions were: MZ3_D332A_BK: 5′-GTCCGCCCATTTGGCTTCGGTGGCGCTATTG-3′; MZ3_D333A_FD: 5′- CAATAGCGCCACCGAAGCCAAATGGGCGGAC-3′; (modified codons are underlined). Mutation was confirmed by sequence analysis of both DNA strands and purified mutant protein was tested for activity against both methyl 4-*O*-methyl-D -glucopyranuronate and *p-*NP acetate under standard assay conditions as described previously^9^.

### Docking

Glide^32,33^ docking was performed with rigid enzyme and flexible substrates. Substrate conformations were generated by an exhaustive enumeration of the minima in the ligand torsion-angle space. The enzyme structure of MZ0003 with the catalytic Ser243 mutated to Ala was used to enable the substrate to adopt a catalytically-relevant conformation without clashes between the substrate and the Ser243. The feasibility of this approach was confirmed by performing control docking simulations with the Ser to Ala mutant of StGE2 which produced an almost identical pose to that experimentally determined from the co-crystal structure, while docking with the WT produced catalytically improbable poses. 3D structures of substrates were either taken from PDB 4g4j (e.g., methyl 4*-O-*methyl-D-glucopyranuronate) or sketched using Maestro in the Schrödinger 2017 Suite. The Ligprep module of Schrödinger was used to generate all possible states of these substrates at a pH range of 7 ± 2. All these substrates post ligand preparation were used for molecular docking studies to understand the binding modes and structural requirements of an ideal substrate.

A grid box was approximately centered around the active site of the MZ0003 structure and the substrates were was treated flexibly while the protein was held rigidly in the docking procedure. Glide uses a several filters in hierarchy to search for possible locations of the ligand in the active-site region of the receptor, which is recognized as “pose” (a complete specification of the ligand: position and orientation relative to the receptor, core conformation and rotamer-group conformations). Then molecular mechanics with generalized Born and surface area (MM-GBSA) scoring function has been used to predict binding free energies.

### Molecular dynamics (MD) simulations

To account for flexibility in the protein during binding, MD simulations were performed in Desmond program in Schrodinger suite for the docked complex of MZ0003 with methyl 4*-O-*methyl-D-glucopyranuronate. The simulations were performed using OPLS_2005 force field in the explicit solvent with the TIP3 model of water^34^. The initial structure of the substrate-enzyme complex was taken from the docking. The water box was chosen to ensure that the entire surface of the complex was covered by the solvent model, and the system was neutralized by adding Na^+^ counter ions to balance the net charges. The system was minimized and pre-equilibrated using the default relaxation routine in Desmond, before a production run of 10 ns. The equations of motion were integrated with 2 fm time step in the NVT ensemble during the simulations, with the temperature 300 K.

### Data availability

The structure coordinates of MZ0003 refined to 1.9 Å are available from the Protein Data Bank as entry 6ehn.

## Electronic supplementary material

Supplementary figures
