# Supplementary material for: Structural insight into a CE15 esterase from the marine bacterial metagenome
Source: Sci Rep. 2017 Dec 8;7:17278. doi: 10.1038/s41598-017-17677-4 (PMC5722869; doi:10.1038/s41598-017-17677-4)
Supplement: Supplementary file 1 — Supplementary figures [file 41598_2017_17677_MOESM1_ESM.pdf]

Supplementary files accompanying manuscript *'Structural insight into a CE15 esterase from the marine bacterial metagenome'*

Concetta De Santi, Osman Gani, Ronny Helland, Adele Williamson

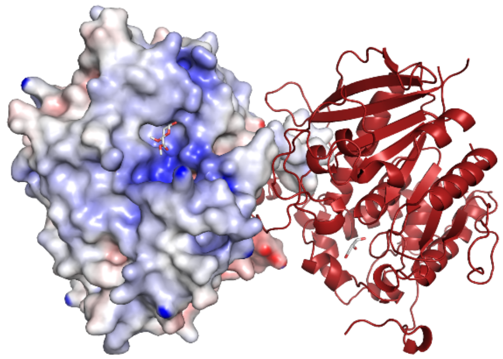

Supplementary figure 1. Dimer of MZ0003 generated based on crystallographic symmetry

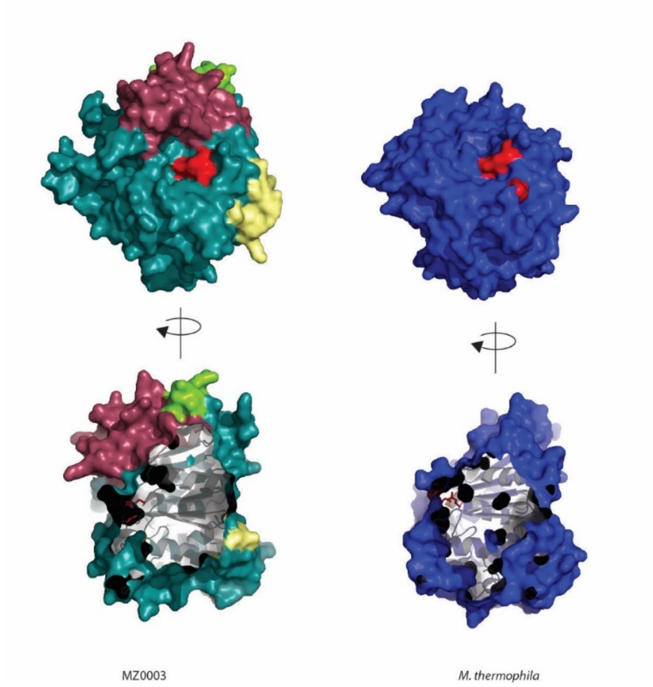

Supplementary figure 2. Surface view of MZ0003 and 4g4g indicating insert regions.

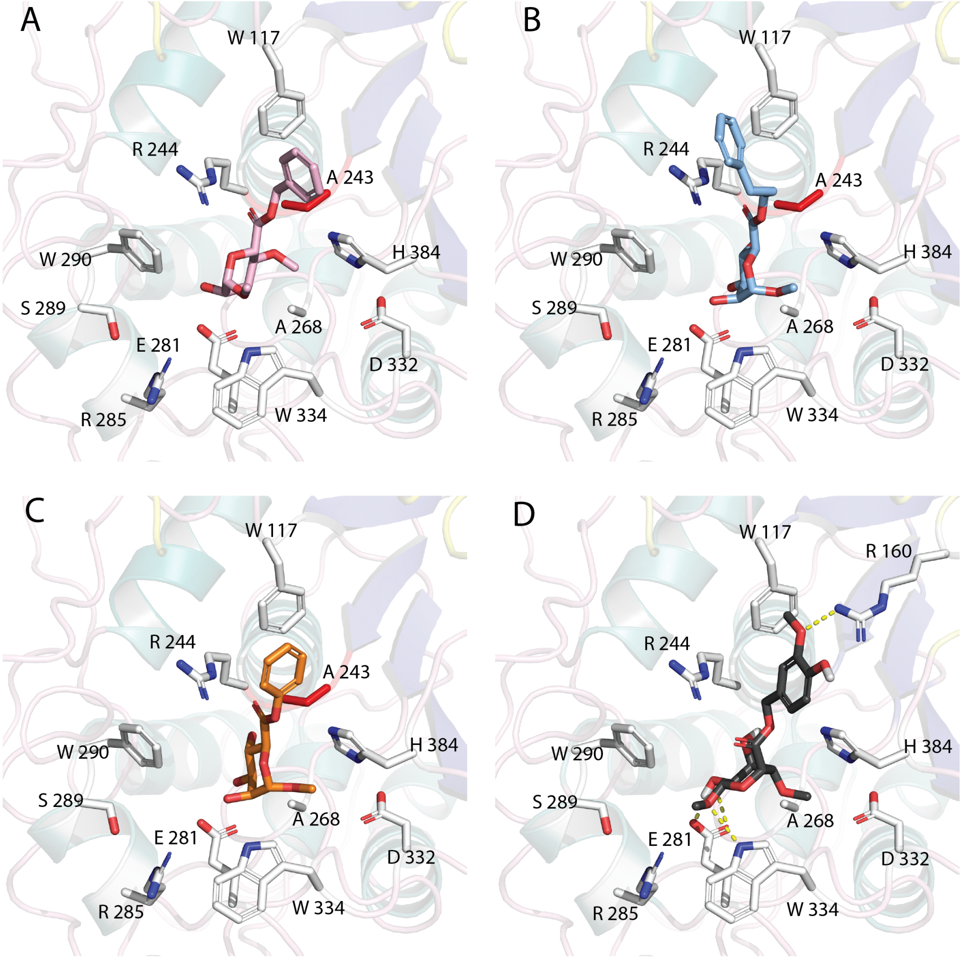

Supplementary figure 3. Binding poses for MZ0003 S243A docked with (A) Benzyl (methyl 4-*O*-methyl-a-D-glucopyranoside) urinate, (B) Phenyl (methyl a-D-glucopyranoside) uronate , (C) Phenylpropyl (methyl a-D-glucopyranoside) uronate and (D) methoxy and hydroxylated Benzyl (methyl a-D-glucopyranoside) uronate

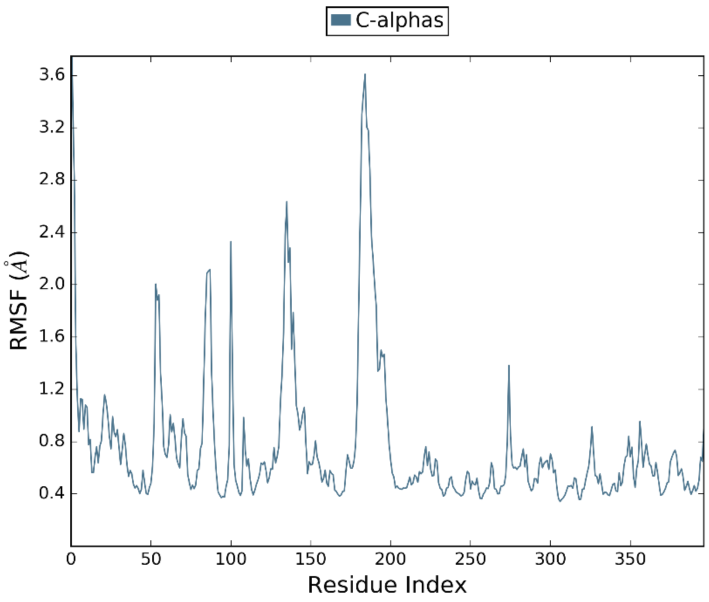

Supplementary figure 4. Protein RMSD of MZ0003 S244A with methyl 4-*O*-methyl-D-glucopyranuronate docked in 0.5M NaCl.
